# Knowledge and attitude of women on the available PMTCT services at the antenatal clinic of the Coast Province General Hospital

**DOI:** 10.11604/pamj.2014.18.4.3839

**Published:** 2014-05-01

**Authors:** Adam Kevin, Marion Mutugi, Peter Wanzala

**Affiliations:** 1 Institute of Tropical Medicine and Infectious Diseases, Jomo Kenyatta University of agriculture and Technology, Nairobi, Kenya; 2 Kenya Medical Research Institute, Nairobi, Kenya

**Keywords:** Prevention of Mother to Child Transmission, antenatal Care, Human Immunodeficiency Virus, Coast Provincial General Hospital, antiretroviral

## Abstract

**Introduction:**

Several high profile events of the last decade have served as catalysts for the now widely available prevention of mother-to-child transmission of HIV services. However, Kenya continues to face challenges in assuring that all women in need of PMTCT services receive the full package.

**Methods:**

A cross sectional survey was undertaken. Systematic sampling method was used for sample selection. Data was collected using pretested structured questionnaires. Data was analyzed in SPSS and Epi Info using bivariate and multivariate logistic regression.

**Results:**

Approximately 75% of participants were seeking PMTCT services in CPGH for the first time, 71% knew of their HIV status. About 95% of participants were satisfied with privacy during testing. Clients who had never delivered in CPGH had a significantly (p<0.001) higher odds compared to those who had previously delivered in CPGH and had their first PMTCT visit. participants who had never lost a pregnancy in CPGH and were in the hospital for the first time were 3 times likely to seek PMTCT services compared to those who had lost a pregnancy in CPGH. There was a significant association between family planning use before pregnancy and first PMTCT.

**Conclusion:**

Participants seeking PMTCT services had poor HIV knowledge; but reported positive experiences and good provider – client relationship. However for a successful PMTCT program in CPGH attention needs to be paid in the patient experiences as they seek other reproductive services.

## Introduction

Mother-to-child transmission (MTCT) is when an HIV-infected woman passes the virus to her baby. This can occur during pregnancy, labor and delivery, or breastfeeding. Without treatment, around 15-30% of babies born to HIV positive women will become infected with HIV during pregnancy and delivery. A further 5-20% will become infected through breastfeeding [1].

In 2009, around 400,000 children under 15 became infected with HIV, mainly through mother-to-child transmission. About 90% of these MTCT infections occurred in Africa where AIDS is beginning to reverse decades of steady progress in child survival [2].

The expected expansion of PMTCT services on the Sub-continent has however, been faced with less than satisfactory utilization. Use of services such as facility delivery and postnatal prophylactic ARV is reported to be between 20% and 47% in many settings [3].

In Kenya, interventions to reduce PMTCT are integrated in Maternal and Child Health (MCH) services in health service delivery institutions. These also include HIV testing during ANC attendance to identify pregnant women living with HIV, maternal and infant antiretroviral treatment, safe obstetrical care, infant feeding counseling and support, and family planning services to delay future pregnancies or prevent unintended pregnancies [4]. Kenya launched its PMTCT program in 2000, but only modest expansion of PMTCT services occurred in the first three years that followed [5].

Despite these low levels of service uptake, there have been limited studies attempting to document reasons for underuse of PMTCT services. The objective of this study was to determine pregnant women's perceptions of service delivery dynamics that are associated with the uptake of PMTCT services.

## Methods

### Study site & Population

The study site was the Coast Province General Hospital a 700 bed hospital located on the coast of the Indian Ocean in the city of Mombasa, south-eastern Kenya with a clientele that are mostly referred from the district hospitals in the province. The hospital serves a population of approximately two million people. The study population was pregnant women who have already enrolled for PMTCT at the antenatal clinic. The Healthcare providers assisted in identifying these women and obtaining consent from them. The research therefore identified the pregnant women's perceptions of service delivery at the health facility that influenced the uptake of PMTCT services.

### Methodology

A cross sectional study was conducted in which 196 women were interviewed. The participants had to meet the inclusion criteria of being pregnant, HIV positive women attending the ANC/PMTCT services at the health facility. Systematic random sampling was done based on the number of HIV positive women attending PMTCT at the antenatal clinic in a period of time. For this case, the researcher identified the total number of women that attend PMTCT services during the morning and early afternoon hours, then the desirable sample size was computed.

Data was collected using pre-tested semi structured questionnaires. Questionnaires were pretested in order to establish if they captured all the variables in this study. The pre-tested questionnaires were administered by the researcher to capture information from the pregnant HIV women on all the variables measured. These patients were identified at the PMTCT clinic; clinicians and nurses in these facilities assisted in identifying these patients. They were only being interviewed after informed consent was obtained. The participants were interviewed after the clinicians and nurses had seen them and referred them to the researcher. The interviews were conducted by the Principal Investigator in enclosed rooms to ensure accuracy and confidentiality.

The quantitative data from the field was coded and double entered into a computer database designed using MS-Access and MS-Excel applications and analysis was done using SPSS Statistical Software and Epi-Info version 6. Fisher's exact test was applied to determine the significance of differences of relative frequencies. Bivariate analysis was performed using Chi square to determine the association between the dependent variable and independent variables. Multivariate logistic regression using backward method was then performed, to calculate adjusted odds ratio for the independent association between late presentation and the independent variables.

### Ethical clearance

Approval was obtained from Kenya Medical Research Institute (KEMRI) Scientific Steering Committees and KEMRI National Ethical Review Committee. Permission to perform the study was obtained from the PMTCT department in CPGH. Prior consent was obtained in accordance with the ethical guidelines. All stake holders were informed about the study. There were rooms in the various facilities that were identified for the interviews to take place so that all the information was confidential Informed consent was sought from the study participants before administration of the questionnaire.

## Results

### General characteristics of the study participants

Majority of the women interviewed (70%) were married while were widowed. Further, majority 43% of the women were unemployed while only 11% had a salaried job. The household monthly income of 6001 to 9000 comprised the income bracket for the majority (21%) however, 43% of the respondents did not have income documented. Majority of respondents (42%) had secondary education while those having college/university education were 6%. The mean age for the respondents was 30 (+5) years while the mean family size was 4(SD 2) persons (range 1- 10) (Table 1).

**Table 1:** Socio-demographic characteristics of respondents

Variable	Category	n(%; 95% CI)
**marital status**		
	Single	22 (15 [9 - 21])
	Married	99 (66 [58 - 74])
	Separated/divorced	11 ( 7 [4 - 13])
	Widow	5 ( 3 [1 - 8])
	Cohabiting	13 ( 9 [5 - 14])
**Employment status**		
	Business	50 (33 [26 - 41])
	Casual	15 (10 [6 - 16])
	Salaried	14 ( 9 [5 - 15])
	Unemployed	67 (45 [37 - 53])
	Others	4 ( 3 [1 - 7])
**Household monthly income**		
	<3000	6 ( 4 [1 - 9])
	3001_6000	18 (12 [7 - 18])
	6001_9000	32 (21 [15 - 29])
	>9000	27 (18 [12 - 25])
	Missing	67 (45 [37 - 53])
**level of education**		
	None	25 (13 [8 - 18])
	Primary	85 (43 [36 - 50])
	Secondary	76 (38 [31 - 45])
	College/University	13 ( 7 [4 - 11])
**Age**		
	Mean (SD)	30[5]
**Family size**		
	Mean (SD)	4[2]

### The association between ANC factors and first PMTCT visit experience

Bivariate Analysis was done to test for the strength of association between categorical variables. Participants who had never delivered in CPGH had a significantly (p<0.001) higher odds by 6 times to use PMTCT services compared to participants who had previously delivered in CPGH. Further participants who had never lost a pregnancy in CPGH and were in the hospital for the first time were 3 times likely to seek PMTCT services in CPGH compared to those who had lost a pregnancy in CPGH. No significant association was identified between duration of time spent on getting to hospital or fare spent to hospital with attending the first PMTCT visit in CPGH. However there was a suggestive trend, although not significant, that the longer it took to get to hospital and hence more fare paid, the more likely it was a participant to utilize PMTCT services for the first time in CPGH (Table 2).

**Table 2:** Association between ANC factors and first PMTCT visit experience

	1^st^ PMTCT visit			95% CI		
	Yes n(%)	No n(%)	OR	Lower CI	Upper CI	P value
**Previously delivered in CPGH**						
Yes	26(17.6)	28(57.1)	(base)			
No	122(82.4)	21(42.9)	6.26	3.09	12.68	<0.001
**Any lost Pregnancy at CPGH**						
Yes	13(8.8)	12(24.5)	(base)			
No	135(91.2)	37(75.5)	3.37	1.42	8.00	0.006
**Transport mode used**						
walking	21(14.0)	3(6.1)	(base)			
car	128(85.3)	46(93.9)	0.40	0.11	1.40	0.150
others	1(0.7)	0(0.0)	(empty)			
**Duration to facility**						
<30 min	20(13.3)	7(14.3)	(base)			
30 min	27(18.0)	14(28.6)	0.68	0.23	1.98	0.474
30 min - 1 hr	96(64.0)	25(51.0)	1.34	0.51	3.53	0.549
1 -2 hrs	5(3.3)	3(6.1)	0.58	0.11	3.10	0.527
2- 3 hrs	2(1.3)	0(0.0)	(empty)			
**Fare spent**						
10_50	38(29.00	23[50.0]	(base)			
50_100	80(61.1)	21(45.7)	2.31	1.14	4.67	0.020
100_200	12(9.2)	2(4.3)	3.63	0.75	17.70	0.111
>200	1(0.8)	0[0.0]	(empty)			
**Time spent in facility**						
<1hr	71(47.7)	16[32.7]	(base)			
1_2hrs	68(45.6)	25[51.0]	0.61	0.30	1.25	0.177
2_3hrs	8(5.4)	7[14.3]	0.26	0.08	0.81	0.021
>3hrs	2(1.3)	1[2.0]	0.45	0.04	5.28	0.526

### The association between utilization of family planning services and first PMTCT visit experience

The FP method used and source of information on FP were not associated with the first PMTCT visit in CPGH, however use of family planning method before was significantly (p=0.001) associated with an increased odds of 6 times of utilizing PMTCT services for the first time in CPGH with use of FP before pregnancy as the reference group (Table 3).

**Table 3:** Association between utilization of Family planning services and first PMTCT visit experience

	1^st^ PMTCT visit			95% CI		
			OR	Lower CI	Upper CI	P value
**Know what is FP**	**Yes n(%)**	**No n(%)**				
Yes	145(96.7)	49(100.0)				
No			empty			
FP method	5(3.3)	0(0.0)				
Condom	33(22.8)	10(20.4)	(base)			
Injection	56(38.6)	14(28.6)	1.21	0.48	3.04	0.681
Implant	19(13.1)	10(20.4)	0.58	0.20	1.63	0.299
Pills	34(23.4)	13(26.5)	0.79	0.31	2.06	0.633
permanent method	1(0.7)	1(2.0)	0.30	0.02	5.29	0.413
Others			0.61	0.05	7.40	0.695
Source of information on FP	2(1.4)	1(2.0)				
CPGH ANC	44(30.3)	22(44.9)	(base)			
other hosp ANC	14(9.70	14(28.60	0.50	0.20	1.23	0.131
Church	16(11.0)	2(4.1)	4.00	0.84	18.97	0.081
Media	24(16.6)	3(6.10	4.00	1.08	14.75	0.037
friends n Family	36924.8)	8(16.)]	2.25	0.90	5.65	0.085
school/college	10(6.9)	0(0.0)	(empty)			
Others			(empty)			
**Family planning use before pregnancy**	1(0.7)	0(0.0)				
Yes	91(60.7)	44(89.8)	(base)			
no	59(39.3)	5(10.2)	5.71	2.14	15.22	0.001

### The association between HIV testing information and first PMTCT visit experience

In regard to HIV information, participants who were not administered with ARVs had a significantly increased odd of 8 times as compared to those who administered ARVs if they were seeking PMTCT services for the first time in CPGH of having received such information. With those who had their HIV results explained as the reference group, participants attending PMTCT for the first time had a significantly decreased odds of 0.1 (95% CI 0.01 – 0.98; p=0.048). Similarly participants who were tested twice had significantly decreased odds of 0.4 seeking PMTCT services for the first time (95% CI 0.21 – 0.79; p=0.008) as compared to those who were tested once. All other HIV related information was not significantly associated with seeking PMTCT services for the first time (Table 4).

**Table 4:** Association between HIV testing information and first PMTCT visit experience

	1^st^ PMTCT visit			95% CI		
	Yes n (%)	No n (%)	OR	Lower CI	Upper CI	P value
**HIV testing information**						
**Confidentiality explained**						
Yes	146(97.3)	46(97.9)	(base)			
No	4(2.7)	1[2.1]	1.26	0.14	11.56	0.838
**ARV administered**						
Yes	111(74.0)	45(95.7)	(base)			
No	39(26.0)	2(4.3)	7.91	1.83	34.13	0.006
**stage of ARV administration**						
First trimester	67(61.5)	21(48.8)	(base)			
second trimester	30(27.5)	16(37.2)	0.59	0.27	1.28	0.182
third trimester	12(11.0)	6(14.0)	0.63	0.21	1.88	0.404
**Pre testing counseling**						
Yes	149(100.0)	45(95.7)				
No	0(0.0)	2(4.3)				
**post-test counseling**						
Yes	143(96.0)	46(97.9)	(base)			
No	6(4.0)	1(2.1)	1.93	0.23	16.45	0.548
**Results explained**						
Yes	148(99.3)	44(93.6)	(base)			
No	1(0.7)	3(6.4)	0.10	0.01	0.98	0.048
**Number of times tested**						
once	102(68.5)	22(46.8)	(base)			
twice	47(31.5)	25(53.2)	0.41	0.21	0.79	0.008

### Independent predictors of undertaking the first PMTCT visit in CPGH

All independent variables identified to significantly associate with the dependent variable at bivariate analysis were considered together in a Multivariate analysis. Factors that were identified as the significant predictors of attending first PMTCT visit in CPGH were; age, previously delivering at CPGH and family planning use (Table 5).

**Table 5:** Multivariate model for the independent predictors of undertaking the first PMTCT visit

	OR	Lower CI	Upper CI	P value	LRT P value
**Age in years**	0.93	0.86	1.01	0.074	0.003
**previously delivered in CPGH**					
Yes	(base)				
No	5.78	2.70	12.38	<0.001	
**Family Planning use**					
Yes	(base)				
No	4.33	1.52	12.35	0.006	

## Discussion

CPGH provides both primary care services and referral services with patients coming for PMTCT services while walking to the facility while others used car transport for up to 2 hours. CPGH is the only referral hospital in Coast province and thus patients from different areas are forced to travel to the hospital to seek both primary care services and referral services. To increase attendance, clinics should aim to be as accessible as possible. Improvements might include providing travel services or changing opening hours. Other researchers highlighted boosting attendance by setting up a Saturday clinic [6].

While women who are HIV-positive should be encouraged to give birth at a clinic, as this reduces the risk of maternal mortality and MTCT, this is often not possible due to the distance between home and clinic. In some clinics, waiting mothers’ shelters provide accommodation for women nearing the end of their pregnancy to ensure they deliver within a healthcare setting [2].

With regard to knowledge and attitudes of mothers on the available PMTCT services there was a significant association between women never having sought reproductive health services in CPGH (e.g. previous delivery, family planning, abortion care etc) and seeking PMTCT services for the first time in CPGH. This may therefore suggests possible bad experiences during other encounters and hence an avoidance for repeat visits. This is evident with women for whom this was not the first pregnancy and had not lost a pregnancy in CPGH being more than 3 times likely to seek the first PMTC visit services in CPGH. This is because this participant group had no prior experience with CPGH services that are normally provided by the hospital e.g. reproductive health services, outpatient services, nutritional services, in-patient services.

These findings are further supported by work done by UNICEF showing that preventing mother-to-child transmission of HIV is reliant on strong healthcare systems and infrastructure. However, in many countries there is a short supply of healthcare workers, which can adversely affect the standard of care and capacity of clinics. Moreover, an inefficient supply of PMTCT drugs or testing kits and the separation of core services can make visits to health clinics prolonged or unnecessary [7]. These negative experiences of health clinics can result in negative attitudes that dissuade pregnant mothers from accessing these key services. A lack of capacity and coordination may also result in gaps in the care delivered to mothers; for example an overworked healthcare worker may fail to deliver a key PMTCT intervention, such as an initial HIV test [8].

Knowledge and Integration of FP, VCT, PMTCT and ART Programs helps the mothers identify existing FP information and services as well as determine their desires and attitudes for family planning within the context of VCT, PMTCT and ART. This integration in addition helps identify operational barriers, gaps, and constraints affecting the provision of family planning [9].

## Conclusion

Independent predictors of utilization of PMTCT services in CPGH were age, previous delivery in CPGH and family planning use highlighting that services provided elsewhere in hospital influence uptake of PMTCT services.

There was poor HIV knowledge especially in those who sought for PMTCT services the first time in CPGH. Although most clients interviewed rated the PMTCT services highly we noted that clients who had previously sort reproductive health services in CPGH were less likely to seek PMTCT services in CPGH suggesting potential bad experiences in the past. There was poor uptake of family planning services in first time users of PMTCT services. Almost all patients were happy with provider-client interaction and privacy provided.
